# Estimation and correlation of serum and salivary glucose and immunoglobulin A levels and salivary candidal carriage in diabetic and non-diabetic patients

**DOI:** 10.34172/joddd.2020.041

**Published:** 2020-11-11

**Authors:** Shruthi S Hegde, Atul P Sattur, Anil Bapu Bargale, Gayathri S Rao, Rajeeth S Shetty, Raghavendra D Kulkarni, Ganavalli S Ajantha

**Affiliations:** ^1^Department of Oral Medicine and Radiology, Srinivas Institute of Dental Sciences, Mukka, Surathkal, Mangalore, Karnataka, India; ^2^Department of Oral Medicine and Radiology, SDM College of Dental Sciences and Hospital, Dharwad, India; ^3^Department of Biochemistry, SDM College of Medical Sciences and Hospital, Dharwad, Karnataka, India; ^4^Department of Oral and Maxillofacial Surgery, Srinivas Institute of Dental Sciences, Mukka, Surathkal, Mangalore, India; ^5^Department of Microbiology, SDM College of Medical Sciences and Hospital, Dharwad, India

**Keywords:** Candida, Diabetes mellitus, Immunoglobulin A, Saliva, Serum

## Abstract

**Background.** A correlation has been noted between diabetes mellitus (DM) and changes in the oral cavity. The present study aimed to estimate, compare, and correlate serum and salivary glucose and IgA levels and salivary candidal carriage in diabetic and non-diabetic individuals.

**Methods.** Eighty-eight subjects were categorized into three groups: group 1 (controlled DM; n=27), group 2 (uncontrolled DM; n=32) and group 3 (non-diabetics; n=29). Serum and salivary glucose levels were estimated by glucose oxidase/peroxidase method, serum and salivary IgA by a diagnostic kit, and candidal colonization by inoculating samples into Sabouraud dextrose agar plate. Statistical analyses were carried out by one-way ANOVA, post hoc Tukey tests, and Pearson’s correlation coefficient.

**Results.** Significant elevation of serum IgA levels was observed in group 2 compared to group 3 and significant decreases in salivary IgA levels in groups 1 and 2. The candidal carriage was significantly higher in group 2 compared to group 3. Serum glucose and salivary IgA levels showed a significant correlation in group 1. There was a positive correlation between serum/ salivary glucose and serum/salivary IgA levels in group 2. In addition, there was a significant correlation between serum glucose and serum IgA levels in group 3.

**Conclusion.** Saliva could be a potential, non-invasive diagnostic tool to estimate glucose levels. The evaluation of salivary components, like IgA, might be useful in diagnosing and managing oral manifestations in diabetic individuals. Elevated salivary glucose levels contribute to elevated candidal carriage, making individuals susceptible to oral candidiasis.

## Introduction

Diabetes mellitus (DM) is a multisystem disorder considered a relative or complete deficiency of insulin release and/or associated resistance to the action or function loss of insulin in target tissues.^1^ The World Health Organization (WHO) has predicted an increase in the number of diabetic patients to >300 million by the year 2025.^2^ In Asia, Indians are at greater risk of developing this disorder; therefore, India is called the “diabetes capital of the world.”^3^ Early screening of DM is necessary for a better prognosis and to avoid clinical complications.^4^ However, to test for hyperglycemia often involves the painful and invasive blood testing procedure, which limits its large-scale applicability. The assessment of saliva has additional advantages over blood due to its non-invasiveness and easier collection with minimal discomfort.^5^ Recently, saliva has been considered a non-invasive tool for diagnosing and managing different disorders by investigators and clinicians.

Saliva consists of locally produced substances plus serum components that can be useful in the diagnosis of various systemic diseases and oral manifestations.^6^ The most crucial specific defense factors of saliva are immunoglobulins (Ig), in which the secretory IgA (sIgA) is predominant, produced by the plasma cells in the salivary glands, which is essential in local (mucosal) immunity.^7^ A fully responsive immunologic system is essential to encounter various infections and toxic agents. Evaluation of saliva constituents is beneficial in the description and management of oral manifestations in diabetic individuals.^8^

The role of *Candida* in the oral manifestations of diabetics is a contentious issue. Salivary qualitative changes, such as the glucose content, influence the candidal carriage in the oral cavity.^9^ Furthermore, sIgA reduces adherence of *Candida albicans* to host surfaces through immune exclusion by binding and aggregating microorganisms within saliva that are then cleared through swallowing.^10^ Very few studies have evaluated the function and composition of saliva in diabetic patients, especially in India; hence, reports are limited to date. Moreover, the results of various studies are inconsistent, indicating the necessity of further investigations. Given these facts, we aimed to estimate, compare, and correlate serum and salivary glucose and IgA levels to assess humoral immune status of individuals and candidal carriage in the saliva of patients with diabetic and in non-diabetic subjects and determine whether glucose levels and components of saliva can be utilized as a non-invasive tool to monitor glycemic control and in the description and management of oral manifestations in diabetics to help advise patients regarding strict diabetes control and take precautions to maintain good oral hygiene to prevent clinical manifestations of candidiasis and its associated morbidity.

## Methods

In the present study, 88 patients were included after a thorough examination based on inclusion and exclusion criteria. A detailed case history was recorded. The patient’s history of the disease duration, glycemic index, family, and personal history were recorded. The participants were briefed about the study and their enrolment, and written consent was obtained.

### Inclusion criteria

Patients selected for controlled and uncontrolled diabetics groups had already been diagnosed with diabetes by the experts/clinicians. The classification of DM^11^ was based on current treatment and provision of blood samples for follow-up purposes and routine check-ups. Those individuals without a history of diabetes and with no symptoms of DM and with a random non-fasting plasma glucose (RNFPG) levels of 80‒120 mg/dL were categorized as the control subject.

### Exclusion criteria

Patients with chronic infections, chronic liver diseases, rheumatoid arthritis, systemic lupus erythematosus, sarcoidosis, myeloma, a history of impaired fasting glucose, pre-diabetics, patients with adverse habits, and those who were on topical or systemic antifungal or steroid therapy, or undergoing treatment for any other illness other than DM and hypertension were excluded.

The patients aged 40‒60 years were classified into three groups: group 1 (controlled diabetics; n = 27) with RNFPG>120 mg/dL and ≤200 mg/dL; group 2 (uncontrolled diabetics; n = 2) with RNFPG >200 mg/dL, and group 3 (non-diabetics; n = 29) with RNFPG 80‒120 mg/dL.

### Estimation of serum and salivary glucose levels

A standardized technique, glucose oxidase peroxidase (GOD-POD) method, was used to estimate serum and salivary glucose by using a spectrophotometer (Systronics spectrophotometer: 2201).^12^ Two mL of peripheral venous blood was collected from each patient under aseptic conditions. Unstimulated saliva was collected by spitting.^9^

### Estimation of serum and salivary IgA

Serum and salivary IgA was measured using a QUANTIA-IgA assay kit (Tulip Diagnostics [P] Ltd., Mumbai, India). QUANTIA-IgA is a turbidometric method based on agglutination reaction for the detection of IgA in serum.^13^

### Sampling of saliva and yeast count assessment

Salivary samples were collected to assess colony-forming units (CFU) of *Candida* by the oral rinse technique using 10 mL of sterile phosphate-buffered saline solution (PBS, pH = 7.4, 0.1 mol/L) for 60 seconds. Sabouraud dextrose agar plates with chloramphenicol (10 mg/mL) were used for inoculation and as an oral rinse after centrifugation. These agar plates were incubated for 48 hours. The growth of *Candida* was confirmed based on smooth, white, or cream-colored buttery colonies, and manual counting of CFU was carried out. To confirm *Candida*colonization, the colony-forming units from random plates were stained with gram staining, and *Candida* growth was identified.^9^

### Statistical analysis

Statistical analysis was carried out with SPSS 21.00. One-way ANOVA was used to compare the three groups, followed by post hoc Tukey tests. Pearson’s correlation coefficient was computed between serum and salivary glucose, IgA, and salivary *Candida* colony-forming units in all the three groups to study the correlation between all the parameters in each group. A regression analysis was used to predict serum glucose based on salivary glucose and serum IgA levels based on saliva IgA in all the three groups. *P*< 0.05 was considered as significant statistically.

## Results

In this study, 88 patients were selected and classified into three groups: 27 in group 1 (controlled DM), 32 in group 2 (uncontrolled DM), and 29 in group 3 (non-diabetics) according to the inclusion and exclusion criteria as mentioned in the methodology.

The subjects consisted of 45 males and 43 females. The respective sex ratio percentages are presented in each study group in Table 1.

**Table 1:** Sex distribution in three groups (controlled DM, uncontrolled DM, and non-DM)

**Groups**	**Male**	**%**	**Female**	**%**	**Total**
Controlled DM (group 1)	14	51.85	13	48.15	27
Uncontrolled DM (group 2)	16	50.00	16	50.00	32
Non-DM (group 3)	15	51.72	14	48.28	29
Total	45	51.14	43	48.86	88

The mean serum glucose level was much higher in group 2 than group 1, with the least in group 3. The differences between these groups were significant statistically (*P* = 0.0001) (Table 2).

**Table 2:** Comparison of three groups with serum glucose levels by one-way ANOVA

**Groups**	**Mean (mg/dL)**	**SD**	**SE**
Controlled DM (group 1)	150.07	38.88	7.48
Uncontrolled DM (group 2)	271.09	59.13	10.45
Non-DM (group 3)	96.69	18.51	3.44
Total	176.49	86.25	9.19
F-value	132.3775		
*P* value	0.00001*		
**Pair-wise comparisons by post hoc Tukey tests**			
Group 1 vs. group 2	*P* = 0.0001*		
Group 1 vs. group 3	*P* = 0.0001*		
Group 2 vs. group 3	*P* = 0.0001*		

*P < 0.05.

The mean salivary glucose level was higher in group 2, followed by group 1 and lower in group 3. However, there were no significant differences between these groups (Table 3).

**Table 3:** Comparison of three groups with salivary glucose levels by one-way ANOVA

**Groups**	**Mean (mg/dL)**	**SD**	**SE**
Controlled DM (group 1)	1.21	1.14	0.22
Uncontrolled DM (group 2)	1.66	1.98	0.35
Non-DM (group 3)	0.86	0.87	0.16
Total	1.25	1.47	0.16
F-value	2.2607		
*P* value	0.1112		
**Pair-wise comparisons by post hoc Tukey tests**			
Group 1 vs. group 2	*P* = 0.5063		
Group 1 vs. group 3	*P* = 0.6714		
Group 2 vs. group 3	*P* = 0.0928		

The mean serum IgA level was higher in group 2 than group 1 and lower in group 3. There was no significant difference between group 1 and group 2 and between group 1 and group 3. However, there was no significant difference between group 2 and group 3 (*P* = 0.0249) (Table 4).

**Table 4:** Comparison of three groups with serum IgA levels by one-way ANOVA

**Groups**	**Mean (mg/dL)**	**SD**	**SE**
Controlled DM (group 1)	753.23	444.32	85.51
Uncontrolled DM (group 2)	802.51	396.00	70.00
Non-DM (group 3)	519.38	406.11	75.41
Total	694.09	428.41	45.67
F-value	3.9433		
*P* value	0.0230*		
**Pair-wise comparisons by post hoc Tukey tests**			
Group 1 vs. group 2	*P* = 0.8924		
Group 1 vs. group 3	*P* = 0.0941		
Group 2 vs. group 3	*P* = 0.0249*		

*P < 0.05.

The mean salivary IgA was much higher in group 1 than groups 2 and 3. There was a significant difference between groups 1 and 2, whereas there were no significant differences between groups 1 and 3 and groups 2 and 3 (Table 5).

**Table 5:** Comparison of three groups with salivary IgA levels by one-way ANOVA

**Groups**	**Mean (mg/dL)**	**SD**	**SE**
Controlled DM (group 1)	166.12	139.45	26.84
Uncontrolled DM (group 2)	86.50	123.98	21.92
Non-DM (group 3)	89.18	113.51	21.08
Total	108.37	128.12	13.66
F-value	2.7551		
*P* value	0.0706		
**Pair-wise comparisons by post hoc Tukey tests**			
Group 1 vs. group 2	*P* = 0.0500*		
Group 1 vs. group 3	*P* = 0.1238		
Group 2 vs. group 3	*P* = 0.9968		

*P < 0.05.

The mean candida CFU was higher in group 2 than group 1 and the least in group 3. There was no significant difference between groups 1 and 2 and between groups 1 and 3; however, there was a significant difference between groups 2 and 3 (*P* = 0.0255) (Table 6).

**Table 6:** Comparison of three groups with log candida scores by one way ANOVA

**Groups**	**Mean (CFU/mL)**	**SD**	**SE**
Controlled DM (group 1)	3.47	2.19	0.42
Uncontrolled DM (group 2)	4.52	1.32	0.23
Non-DM (group 3)	3.20	2.27	0.42
Total	3.76	2.01	0.21
F-value	3.9638		
*P* value	0.0226*		
**Pair-wise comparisons by post hoc Tukey tests**			
Group 1 vs. group 2	*P* = 0.1030		
Group 1 vs. group 3	*P* = 0.8591		
Group 2 vs. group 3	*P* = 0.0255*		

*P < 0.05.

The correlation among serum and salivary glucose and IgA levels and candida CFU in group 1 is presented in Table 7. However, there was a significant and inverse correlation between salivary IgA and serum glucose levels in group 1 (*P* = 0.5964).

**Table 7:** Correlations among serum glucose, salivary glucose, serum IgA, salivary IgA levels and log candida scores in controlled DM (group 1)

**Variables**	**Serum glucose**	**Salivary Glucose**	**Serum IgA**	**Salivary IgA**	**Log candida**
Serum glucose	-				
Salivary glucose	r = -0.4185	-			
Serum IgA	r = -0.2978	r = 0.4470	-		
Salivary IgA	r = -0.5964*	r = -0.0142	r = 0.2127	-	
Log candida	r = -0.0836	r = -0.2206	r = -0.2016	r = 0.0980	-

*P < 0.05.

The correlation between serum and salivary glucose and IgA levels and candida CFU in group 2 is shown in Table 8. There was a significant and positive correlation between serum and salivary glucose (*P* = 0.3677) and between serum glucose and salivary IgA levels (*P* = 0.4763).

**Table 8:** Correlations among serum glucose, salivary glucose, serum IgA, salivary IgA levels and log candida scores in controlled DM (group 2)

**Variables**	**Serum glucose**	**Salivary Glucose**	**Serum IgA**	**Salivary IgA**	**Log candida**
Serum glucose	-				
Salivary glucose	r = 0.3677*	-			
Serum IgA	r = 0.3192	r = 0.3368	-		
Salivary IgA	r = 0.4763*	r = 0.2964	r = 0.1601	-	
Log candida	r = 0.1390	r = 0.2699	r = -0.1319	r = 0.3574	-

*P < 0.05.

The correlation between serum and salivary glucose and IgA levels and candida CFU in group 3 is presented in Table 9. However, there was only a positive correlation between serum glucose and IgA levels.

**Table 9:** Correlations among serum glucose, salivary glucose, serum IgA, salivary IgA levels and log candida scores in controlled DM (group 3)

**Variables**	**Serum glucose**	**Salivary Glucose**	**Serum IgA**	**Salivary IgA**	**Log candida**
Serum glucose	-				
Salivary glucose	r = -0.0231	-			
Serum IgA	r = 0.5800*	r = 0.0609	-		
Salivary IgA	r = -0.3383	r = 0.3251	r = -0.2010	-	
Log candida	r = 0.1186	r = -0.0170	r = 0.0557	r = 0.0317	-

*P < 0.05.

**Figure 1:**
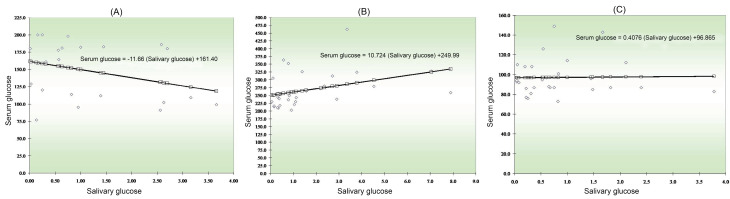
Regression line between serum and salivary glucose in **(A)** controlled DM (Group I), **(B)** uncontrolled DM (Group II) and** (C)** non-diabetics (Group III).

The regression line between serum and salivary glucose levels in group 1 showed that as the salivary glucose levels increased, serum glucose levels decreased in group 1 (Figure 1A). In group 2, the regression line between serum and salivary glucose showed that as the salivary glucose levels increased, serum glucose levels decreased, too (Figure 1B). In group 3, the regression analysis between serum and salivary glucose levels showed that as the salivary glucose level increased, there was no significant increase in serum glucose levels (Figure 1C).

**Figure 2:**
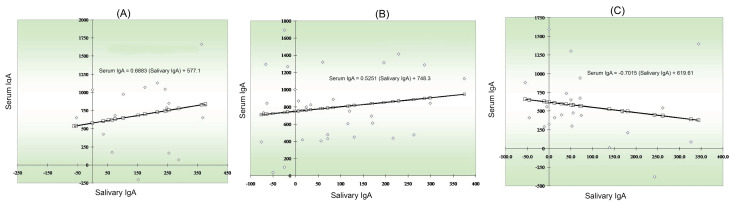
Regression line between serum and salivary IgA in **(A)** controlled DM (Group I), **(B)** uncontrolled DM (Group II) and** (C)** non-diabetics (Group III).

A regression analysis between serum and salivary IgA levels in group 1 showed that as the salivary IgA level increased, serum IgA levels increased, too (Figure 2A). Regression analysis between serum and salivary IgA levels in group 2 showed that as salivary IgA levels increased, serum IgA levels increased, too (Figure 2B). A regression analysis between serum and salivary IgA levels in group 3 showed that as the salivary IgA levels increased, serum IgA levels decreased, too (Figure 2C).

## Discussion

DM is the most common endocrine disorder characterized by a lack of cells’ ability to use glucose. Glucose levels significantly change in DM.^14^ DM has been reported to change the composition and function of saliva as a result of changes in oral hemostasis.^15^

Normal salivary glucose levels do not affect the health of the oral cavity or enhance microbial growth significantly. However, increased salivary glucose favors the microbial proliferation, and enhanced colonies are seen on teeth and oral mucous membranes. Glucose is a nutrient for *Candida* colonization; thus, suppressing the phagocytic activity of neutrophils, which further enhances colonization with possible consequences, can be anticipated because of the increased glucose levels in the saliva of diabetes.^15^

A higher level of immunoglobulins in gingival tissue might be a protective mechanism against the increased bacterial infection in diabetics.^16^ The altered immune response might be the principal causative factor for various oral manifestations of DM. In the present study, most individuals were male in groups 1 and 3, whereas in group 2, males and females had an equal ratio. In the present analysis, the mean serum glucose levels were much higher in uncontrolled DM (271.09 ± 10.45 mg/dL) compared to controlled DM (150.07 ± 7.48 mg/dL) and least in non-diabetics (96.69 ± 3.44 mg/dL). The differences between these groups were significant (*P* = 0.0001).

Furthermore, in the present study, glucose levels in unstimulated saliva of diabetics and non-diabetics were analyzed in each group to verify whether salivary glucose levels follow the serum glucose levels in DM. The mean salivary glucose was found to be higher in uncontrolled DM (1.66 ± 0.35 mg/dL) followed by controlled DM (1.21 ± 0.22 mg/dL) and non-diabetics (0.86 ± 0.16 mg/dL), consistent with previous studies.^9,17^ This finding shows that salivary glucose levels follow a threshold mechanism. Elevated glucose levels in blood above threshold lead to the seepage through the glands’ basement membrane, mainly the parotid gland.^18^ Salivary samples that had been collected in this study signified total oral fluids, thus revealing the glucose levels not only because of seepage through the basement membrane of salivary glands but possibly also through the gingival crevicular fluid. However, in this study, the salivary glucose levels were higher in uncontrolled DM than controlled DM and non-diabetics; however, there were no significant differences between the groups.

Serum and salivary glucose level analysis in three groups revealed significant correlation in uncontrolled DM only, but not in the controlled and non-diabetic subjects, consistent with a study by Sashikumar et al.^9^ Further investigation is necessary to confirm and support whether this reproduces the sensitivity of the test used in the present study or other factors. The regression line between serum and salivary glucose levels showed that as the salivary glucose levels increased, serum glucose levels decrease in controlled DM and uncontrolled DM. In contrast, in non-diabetics, as salivary glucose levels increased, there was not much significant increase in serum glucose levels. The glucose levels in saliva thus closely reflect the serum levels of glucose in the blood and can be used to monitor glycemic control as a reliable non-invasive tool in diabetics.

In the present study, the mean serum IgA level was higher in the uncontrolled DM group (802.51 ± 70.00 mg/dL) compared to the controlled DM (753.23 ± 85.51 mg/dL) and non-diabetic (519.38 ± 75.41 mg/dL) groups. There was no significant difference between the controlled DM and uncontrolled DM groups and between controlled DM and non-diabetic groups. However, there was a significant difference between uncontrolled DM and non-diabetic groups (*P* = 0.0249). There was no significant correlation between serum glucose, serum IgA, and salivary glucose levels in controlled DM and uncontrolled DM groups, whereas, in the non-diabetic group, there was a positive correlation between serum glucose and IgA levels.

Gill et al^19^ and Cheţa et al^20^ studied serum IgA levels in diabetics and healthy individuals. They reported significantly higher serum IgA levels in diabetics than healthy individuals. This indicates the presence of some amount of systemic infection in diabetics. That is why the body’s natural immune system tries to synthesize more immunoglobulins to overcome or minimize systemic infections. Hence, increased serum immunoglobulins can be used as one of the parameters of judging the presence of systemic infections.

In this study, mean salivary IgA levels were much higher in the controlled DM group (166.12 ± 26.84 mg/dL) compared to the uncontrolled DM (86.50±21.92 mg/dL) and non-diabetic (89.18 ± 21.08 mg/dL) groups. There was a statistically significant difference between controlled and uncontrolled DM groups, whereas there was no significant difference between controlled DM and non-diabetic groups and between uncontrolled DM and non-diabetic groups.

There was no significant correlation between salivary IgA and serum IgA and salivary glucose levels in controlled DM and uncontrolled DM groups. However, there was a significant and inverse correlation between serum glucose and salivary IgA levels in the controlled DM group, whereas, in the uncontrolled diabetic group, there was a significant and positive correlation between serum glucose and salivary IgA levels. In non-diabetics, there was no significant correlation between serum IgA and salivary IgA levels, and serum and salivary glucose levels.

The regression line between serum IgA and salivary IgA levels in controlled DM and uncontrolled DM groups showed that salivary IgA levels increased, serum IgA levels increased, too, whereas, in non-diabetics, as salivary IgA levels increased, serum IgA levels decreased. Mata et al^21^ reported variations in salivary compounds in DM patients. These alterations in the whole saliva in patients with DM were not the same in different studies, possibly due to differences in the collection of samples and study design.

In the present study, there was a marked elevation in salivary IgA levels in controlled DM individuals than uncontrolled DM and non-diabetics. Yavuzyilmaz et al^22^ reported higher salivary IgA levels in diabetic patients than controls. They mentioned a possible cause in these patients; it could be associated with factors like calculus and greater accumulation of bacterial plaque. Hyperglycemia is seen in DM, which reduces phagocytosis by granulocytes and helps the colonization of microorganisms. In uncontrolled DM, ketoacidosis is a significant complication, which might disrupt granulocytic migration to the injury site and reduce phagocytosis. Hyperglycemia also alters the neutrophil function and affects chemotaxis. Thus, elevated IgA levels in diabetic patients might be because of the occurrence of candidal species and also the presence of a humoral response.^23^ In the immune system, compensatory mechanisms also influence positive humoral responses and an increase in salivary IgA levels. Our salivary IgA data are contradictory to other studies.^19,24^ These discrepancies could be because of variable saliva sample collection conditions, the stage and status of the disease, and the metabolic control. In the present study, the salivary IgA levels were lower in the uncontrolled DM group than controlled DM and non-diabetic groups, consistent with a study by Bhuyan et al.^25^ The decrease in salivary IgA level in diabetes might be associated with reduced local immune response in the form of sIgA. This could be one of the predisposing factors that make diabetic patients more susceptible to oral infections. It was also detected that salivary IgA levels further reduced in patients with uncontrolled diabetes compared to controlled diabetics, making the uncontrolled diabetics more susceptible to infections. Therefore, effective control of diabetes is essential to minimize infections in the oral cavity. Salivary IgA levels should be evaluated in these patients from time to time to control diabetes and infections.

In the present study, the mean candida CFU was higher in diabetic subjects with uncontrolled DM (4.52 ± 0.23 CFU/mL) compared with those with controlled DM (3.47±0.42 CFU/mL) and non-diabetic subjects (3.20 ± 0.42 CFU/mL), consistent with previous studies.^26-28^ Increased salivary glucose levels increase adherence of candida to buccal epithelial cells. Glycosylation products with proteins in tissues, which are chemically reversible, formed by glucose in saliva during hyperglycemic episodes result in glycosylation of the products accumulating on buccal epithelial cells, leading to increased amounts of available receptors for *Candida*.^29,30^

Reduced candidacidal activity in neutrophils is mainly seen in the presence of glucose and raised candidal carriage in the oral cavity because of a reduction in the salivary flow; many other factors, too, play a role in DM.^31,32^ However, in this study, there is no significant difference between controlled DM and uncontrolled DM and between controlled DM and non-diabetics. However, there was a significant variation between uncontrolled DM and non-diabetic subjects (*P* = 0.0255). However, no significant correlation was observed between candida and serum and salivary glucose levels, and serum and salivary IgA levels in the study groups.

According to this study, *Candida* colony formation in the oral mucosa was much more significant in DM than normal individuals, as observed by other studies.^26-28^ It was also demonstrated that DM patients were predisposed to the colonization of opportunistic *Candida albicans* subclinically, without any clinical lesion of oral candidiasis.^33^

## Conclusion

The present study showed a concurrent rise in glucose levels in serum and saliva of DM cases. Therefore, salivary glucose levels could be a potentially non-invasive diagnostic tool in monitoring glycemic status in diabetic individuals. Evaluating salivary constituents, such as IgA, could be beneficial in diagnosing and managing oral findings in DM. Predisposition to oral candidiasis is likely to be present in diabetic patients due to a rise in salivary glucose levels, contributing to increased candidal carriage.

Salivary glucose levels can be considered a fast and cost-effective method for routine investigations to evaluate oral candidal carriage. This would help counsel patients to take precautions to maintain better oral hygiene and control diabetes strictly to avoid oral candidiasis and its complications.

## Authors’ Contributions

SSH, APS, and ABB were involved in designing the study, collection of data, data acquisition, data analysis, manuscript preparation, drafting, and revision of manuscript, editing, and final approval. The rest of the authors were involved in drafting and final approval of the manuscript.

## Acknowledgments

We are grateful to Dr S B Javali and Dr M V Muddapur for their valuable help in statistical analysis.

## Funding

We wish to acknowledge support from Colgate-Palmolive (India) Ltd. for the present study.

## Competing Interests

The authors declare no competing interests with regards to the authorship and/or publication of this article.

## Ethics Approval

The ethical clearance was obtained from the institutional ethics committee.
